# Cytological, Histochemical, and Ultrastructural Study of the Human Fetal Spleen of Various Gestational Age With Future Implications in Splenic Transplantation: An Anatomical Perspective

**DOI:** 10.7759/cureus.18911

**Published:** 2021-10-19

**Authors:** Arpan Haldar, Manisha Gaikwad, Apurba Patra, Soumya Chakraborty

**Affiliations:** 1 Anatomy, All India Institute of Medical Sciences, Deoghar, Deoghar, IND; 2 Anatomy, All India Institute of Medical Sciences, Bhubaneswar, Bhubaneswar, IND; 3 Anatomy, All India Institute of Medical Sciences, Bathinda, Bathinda, IND; 4 Anatomy, Employees’ State Insurance Corporation’s (ESIC) Post Graduate Institute of Medical Science and Research Medical College and Hospital, Kolkata, IND

**Keywords:** fetus, splenic primordium, immunohistochemistry, gestational age, lymphoid organ

## Abstract

Purpose: The spleen is a fist-sized largest lymphoid organ located in the left hypochondrium. It has a unique location, embryological and histological structure that differs significantly from other lymphoid organs. The present work was undertaken to study the microscopic and ultramicroscopic histogenesis patterns of the spleen in relation to gestational age.

Methods: The splenic tissue of nine aborted fetuses of various gestational ages was studied. For cytology study, special stains like Masson’s trichrome, Periodic Acid-Schiff, and Reticulin were used; immunohistochemical staining was performed with triple antibodies (C-myc, Ki-67, and Ber-H2); and for ultrastructure study, aluminum mounted specimens coated with gold and argon gas were observed under scanning electron microscopy (SEM).

Results: Microscopy and immunohistochemistry showed the developmental changes in the spleen from the emergence of the primordium to the end of the embryonic period in all stages of fetogenesis. The spleen primordium of a fetus at the developmental stage of the primary vascular reticulum was seen in the first trimester fetuses. The primordium is comprised mainly of mesenchymal tissue; numerous lymphocytes invading the area surrounding the central artery forming the periarterial lymphoid sheaths (PALS) were seen surrounded by venous sinuses in the early second trimester fetuses.

Conclusion: The organizational changes in the reticuloendothelial system and microstructure of the spleen during fetogenesis are very crucial to achieving adult morphology in the future. Histogenesis of the fetal spleen follows a multistep process depending upon the gestational age. Any deviation from normalcy may lead to structural and functional abnormality later in life.

## Introduction

The skeletal framework of the spleen resembles a large lymph node, primarily acts as a blood filter. It plays important role in regard to red blood cells (erythrocytes) and the immune system. The mononuclear phagocyte system is responsible for taking apart the old red blood cells and recycling them. It also breaks down the heme portion of the hemoglobin into its constitutive amino acids [1]. The spleen is surrounded by a capsule that's composed of smooth muscle, dense tissue, and elastic fibers. The capsule sends septa into the splenic parenchyma, which divides the parenchyma into irregularly spaced trabeculae. These trabeculae contain blood vessels, lymphatics [2]. The structure of red pulp and white pulp varies among vertebrates and non-vertebrates. The cell population of splenic tissue also varies at different stages of development which is represented by their staining property through immunohistochemistry. Splenic chordal macrophages have many dendritic cytoplasms which are immune positive. The spleen primordium of a fetus at the developmental stage of the primary vascular reticulum can be seen which is mainly comprised of mesenchymal tissue. These minute details about the cellular organization of the vascular reticulum are really crucial during segmental resection of the spleen in adult age due to splenic rupture. Many researchers [3,4] have studied the histogenesis and morphogenesis of spleen parenchyma but none of them have described the ultrastructure of the splenic vascular reticulum concerning the gestational age. Also, data showing the immunoelectron microscopy of the human fetal spleen, which can be in future studies correlated with antibodies present in the spleen, are sparse. Such knowledge will be of immense help in deciding the future course of fetal spleen transplantation. With such a background, the present study was undertaken to look into the cytological, immunohistochemical, and ultrastructural characteristics of developing spleens of various gestational ages and their future implications in splenic transplantation.

## Materials and methods

The splenic tissue of aborted human fetuses of first, second, and third trimesters of gestation, which did not show any evidence of external morphological abnormality, was collected from the Department of Gynaecology and Obstetrics, All India Institute of Medical Sciences, Bhubaneswar after therapeutic abortion, which was used in this study after approval of the Institutional Ethical Committee of Human Research (IEC) and Institute Research Board (IRB) of AIIMS Bhubaneswar (T/IM-NF/Anatomy/18/84). They were collected after obtaining written informed consent of the legal guardian accorded with institutional guidelines, which is maintained in the Fetal Death Register in the Department of Anatomy, AIIMS Bhubaneswar.

For cytological study

The fetuses were dissected by dissecting microscope and the splenic tissue of 2 mm size were immediately fixed in 10% formalin for 24 hours. After fixation by formalin, the tissues were processed for routine histological procedures using special stains such as Masson’s trichrome and reticulin stain, and then photomicrographs were taken by the camera using a microscope adapter.

For immunohistochemical study

Three-millimeter sections of splenic tissues were fixed in 10% neutral buffered formalin (NBF) for seven hours followed by routine processing and paraffin embedding. Unstained sections were immersed in DAKO Target Retrieval Solution and heated for antigen retrieval from tissues. Deparaffinized sections were incubated with 3% hydrogen peroxide for five minutes, which block and inhibit the endogenous enzymes to avoid producing non-specific binding of antigen on the cell surface, followed by sequential 10 minutes incubation in xylene and alcohol. The sections were put in a pressure cooker for 20 minutes (heat-induced epitope retrieval, HIER) to unmask the antigen epitopes in order to allow the antibodies to bind which was followed by a biotinylated link antibody and peroxidize-labeled streptavidin, which augmented the antigen expression on the tissue surface. A modified labeled avidin-biotin immunohistochemical staining was performed with triple antibodies C-myc, Ki-67, and Ber-H2 with the help of an immunoperoxidase kit on DAKO auto Stainer. Staining was completed after a 10 minutes incubation with DAKO 3,3’-diaminobenzidine (DAB) chromogen, which was used as a signal enhancer.

For the ultramicroscopic or ultrastructural study

The splenic tissue was cut in 2 × 2 mm^2^ sizes and was put in primary fixative (Karnovsky's fixative)-2.5% glutaraldehyde + 2% paraformaldehyde in 0.1 M PB (pH 7.4) for 12 hr at 4 °C followed by secondary fixation - 1% OsO_4_ for 1 hour at 4 °C.

The tissue was then dehydrated in ascending grades of alcohol at 4 °C and kept in acetone at room temperature followed by processing with xylene. Epoxy resin infiltration was done with descending toluene: ascending resin ratio for better infiltration of tissue.

The resin block was then cut by knife boat into 50-70 nm sections by ultramicrotome. Double staining was done by aqueous uranyl acetate and alkaline lead citrate. The slides were then viewed under transmission electron microscopy (TEM) and photomicrographs were taken.

## Results

Microscopic findings

First Trimester

**Figure 1:**
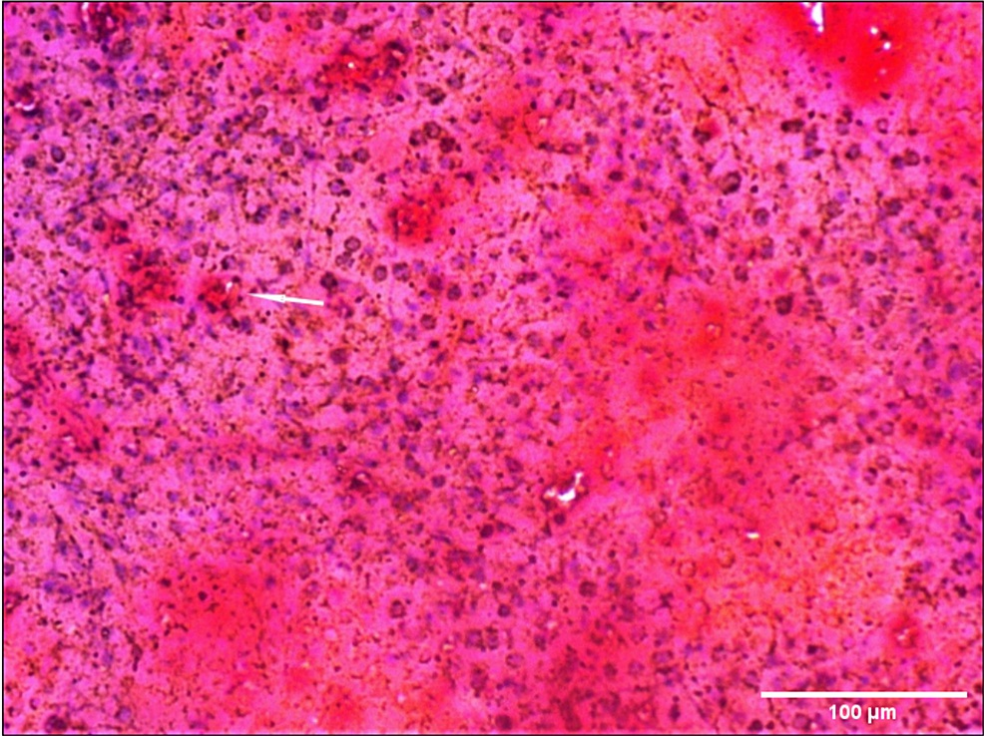
Photomicrograph (10×) showing lymphocytes surrounded by newly forming blood vessels seen in Mason’s trichome stain (fetal red pulp).

The appearance of splenic capsule, reticular framework developed, and lymphoblasts were seen in scattered groups. Lymphocytes surrounded by newly forming blood vessels can be seen in Masson’s trichome stain (fetal red pulp; Figure 1).

Second Trimester

Capsule thickened, reticular fibers formed a skeletal network, sinusoids developed, and vascularity increased. Characteristic features of red and white pulp were seen exclusively. White pulp is compactly arranged lymphocytes with an eccentric arteriole or the periarteriolar lymphatic sheaths (PALS). Red pulp was visualized as sinusoids and RBCs in between white pulp.

Third Trimester

The structure is similar to the adult spleen, thickened well-defined capsule with trabeculae dividing parenchyma into lobules. Characteristics of red and white pulp became more prominent.

Immunohistochemical study

**Figure 2:**
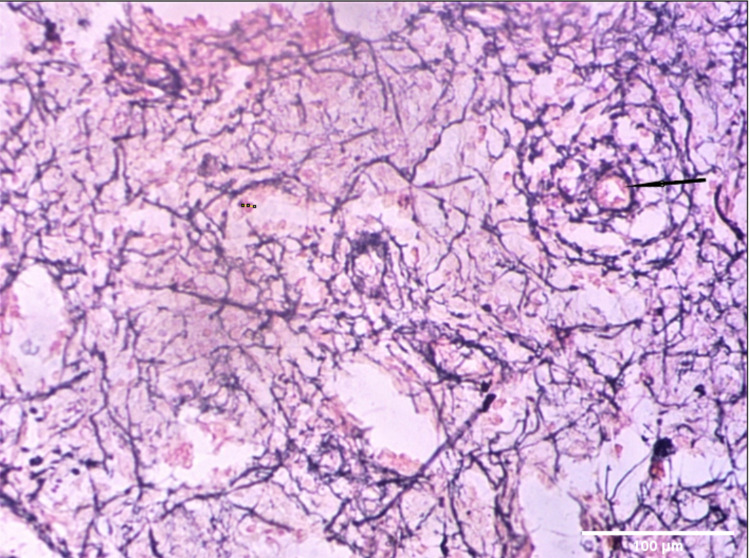
Photomicrograph (40×) showing scattered reticular fibers seen in the reticular stain. White pulp is visualized with a brownish tinge suggestive of immune positivity.

Splenic chordal macrophages showed many immune positive dendritic cytoplasm. Sinus endothelial cells were demonstrated by their expression of the antigen. Erythrophagocytosis by rounded macrophages was seen apparently within sinuses in a fetal spleen. Scattered mid and late normoblasts with characteristic condensed nuclear chromatin were also seen in the fetal spleen. Scattered reticular fibers and white pulp with a brownish tinge suggestive of immune positivity were seen in reticular-stained specimens (Figure 2).

**Figure 3:**
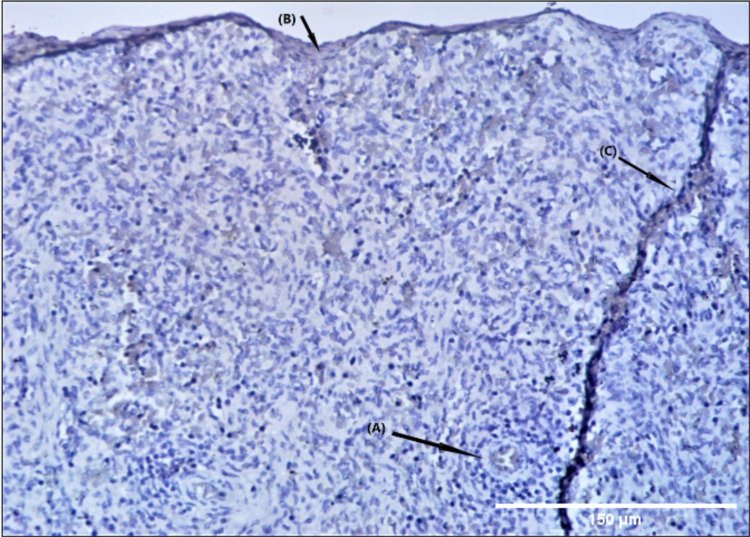
Photomicrograph (20×) showing lightly stained "island" with a central artery in the mesenchymal reticulum, which becomes more prominent in C-myc antibody stain (A). Splenic capsule (B) sending trabeculae (C) can also be seen.

In later weeks of gestation, the immune positive splenic capsule was also visualized along with splenic lobulations. The fetal spleen was characterized by the presence of splenic lobule in the early stage of development (first trimester). A lightly stained "island" with a central artery was seen in the mesenchymal reticulum. These lightly stained "islands" around the central artery become more prominent with C-myc antibody stain (Figure 3).

**Figure 4:**
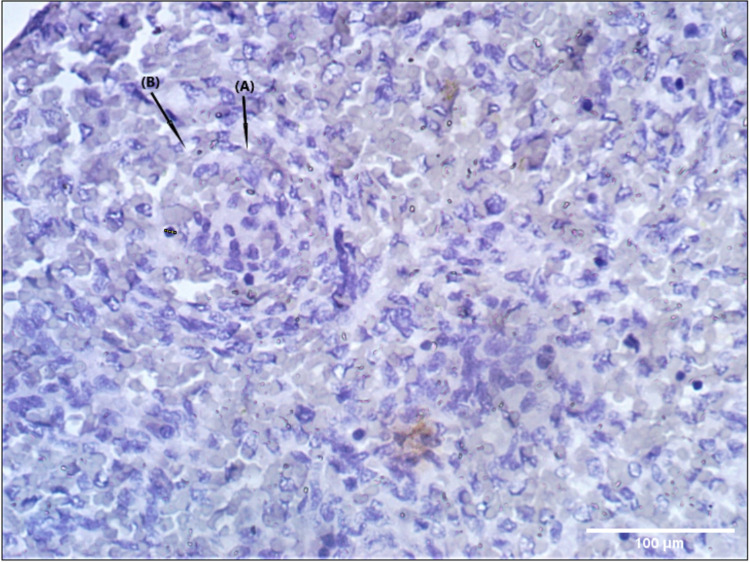
Photomicrograph (40×) showing central artery in developing white pulp displaying relatively high endothelial cells and a continuous basal lamina (A). The artery is surrounded by myoblasts with large, lightly stained, ovoid, or kidney-shaped nuclei (B) in Ki-67 antibody stain.

These “islands” were surrounded by the developing red pulp with numerous venous sinuses. At some places, the basal lamina of these venous sinuses was discontinuous at some places, indicating open communication to the reticulum. The central artery in developing white pulp exhibiting relatively high endothelial cells with a continuous basal lamina was seen in the Ki-67 antibody stain. The artery was surrounded by myoblasts with large, lightly stained, ovoid, or kidney-shaped nuclei (Figure 4).

Smooth muscle similar to the bundles of myofilaments in oval or fusiform dense patches was also seen. These dense patches were more aggregated near the basal lamina between the myoblasts. Precursors of fibroblastic reticulum cells, normoblast, and thrombocytes were seen in the developing red pulp.

**Figure 5:**
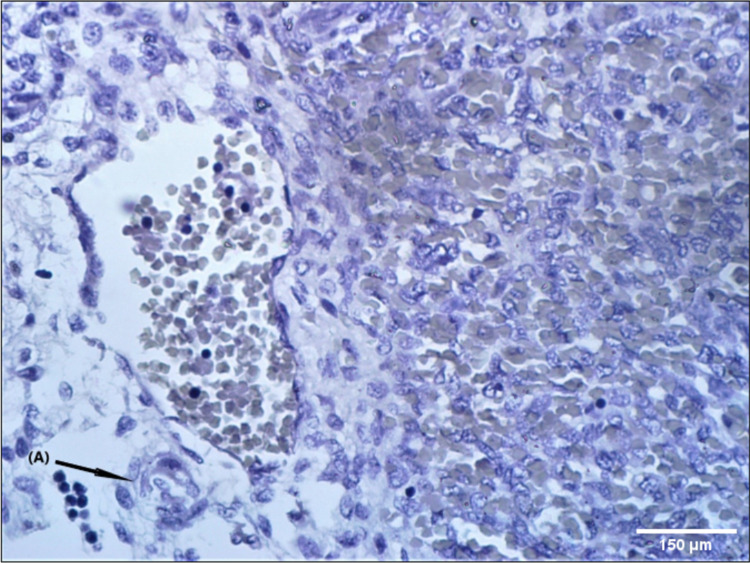
Photomicrograph (40×) showing numerous lymphocytes invading the area around the central artery forms the periarterial lymphoid sheaths surrounded by venous sinuses in CD30 antibody stain (A).

Numerous lymphocytes were seen infiltrating and surrounding the central artery forming the PALS and surrounded by venous sinuses in CD30 antibody stain (Figure 5).

This process of infiltration obscures the splenic lobules. The trabecular arteries and veins are embedded in dense connective tissue. The periarterial lymphoid sheath has widened remarkably. An eccentric accumulation of lymphoid cells indicates the initial formation of a follicle, while the B-cell region in the white pulp can be seen.

Ultramicroscopic study

The splenic primordium was seen in the development stage of its primary vascular reticulum. It was mainly composed of mesenchymal tissue. The specific organization of the spleen was not yet identifiable. The area under the capsule was infiltrated by itinerant cells, chiefly erythrocytes, and their precursors. Whereas, the central areas were densely packed by argyrophilic reticular fibers. Mesenchymal cells were with an irregularly shaped euchromatic nucleus and had a narrow rim of marginal heterochromatin. The cytoplasm of these cells contained a few cisternae of the rough endoplasmic reticulum. The cells were interconnected by desmosomes with reticular fibers and macrophages in close vicinity.

**Figure 6:**
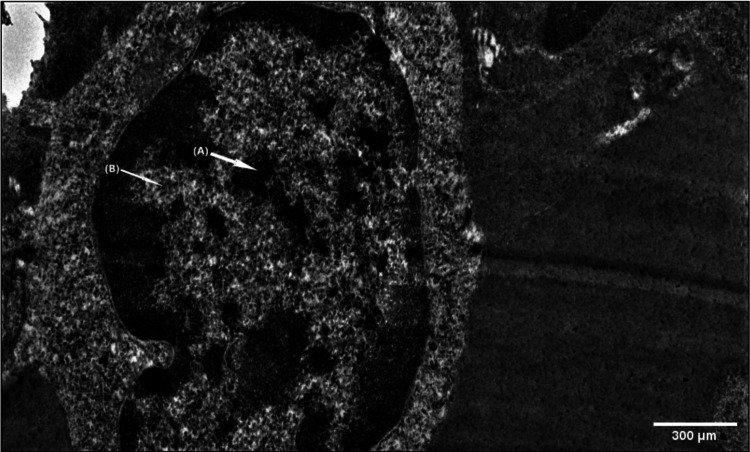
Scanning electron microscopy showing intercellular spaces between lymphocytes (A). The cytoplasmic processes form an interwoven root-like network (B).

Lymphoid cells in the PALS have morphological criteria of T-cell precursors with a blunt lobular nucleus and broad cytoplasmic rim containing mitochondria, polyribosomes, and few lysosomal structures near the nuclear notch. Precursor of an interdigitating cell (IDC) was seen in the PALS. The cytoplasm was electron translucent and possessed short processes. It contained a single lamella of the endoplasmic reticulum. The nucleus showed an irregular shape and a narrow margin of heterochromatin. Follicular dendritic reticulum cell (FDRC) precursor was also seen in electron microscopy. The nuclei were elongated and irregular. The cytoplasmic processes form an interwoven root-like network (Figure 6).

## Discussion

Developmental changes in the microscopic features of the spleen were studied by few researchers earlier but most of them used commonly available hematoxylin and eosin (H&E) for staining purposes, moreover, a simple microscopy technique was used to visualize the structures of the fetal spleen. These are the major flaws in the detailed microscopic study of the spleen.

According to Hamilton et al. [5], at around the 4th-5th month of gestation, mesenchymal cells rapidly multiply and differentiate into erythroblasts, myeloblasts, and megakaryocytes. In the present study, splenic primordium is mainly comprised of mesenchymal tissue with primary vascular reticulum in the early embryonic stage. The specific splenic tissue organization was not attained at this stage. Features like subcapsular region densely infiltrated by itinerant cells (predominantly erythrocytes and precursors) and central areas with densely packed argyrophilic reticular fibers were seen [6,7].

Fetal spleen as an active site for the production of hematopoietic progenitors and normoblasts is a matter of debate, while most of the textbooks of pathology describe the spleen of mid-gestation as a site for erythropoiesis and granulocytopoiesis. Calhoun et al. [8] showed that unlike the spleen of fetal rats, the human fetal spleen does not normally function as an active site for granulocytopoiesis or erythropoiesis, nor is it an active site for the production of granulocyte colony-stimulating factor (G-CSF). According to Copenhaver et al. [9], the fetal spleen participated in the development of lymphocytes, erythrocytes, and granular leukocytes and erythropoietic function ceased at about the eighth month.

While in the present study, we have observed the hemopoietic cells throughout all the stages. Precursor of a fibroblastic reticulum cell, a normoblast, and a thrombocyte in the forming red pulp was seen. Precursor of a monocyte (rarely seen in the developing spleen) was also noticed. The nucleus of these cells was a kidney-shaped euchromatic nucleus with a prominent rim of marginal heterochromatin. Whereas, the cytoplasm contained polyribosomes and numerous small granules.

Van Furth et al. [10] observed lymphoid blast cells having larger chromatin and poor nucleus in the white pulp, while red pulp showed distinct cytoplasmic pyrinophilic cells with a round nucleus in the early stage. Later on, the fetal spleen showed an increase in primary lymphocytes.

Formation of the skeletal framework of the spleen takes place during fetogenesis and it is a multistage process. Various textbooks and researchers have described the histogenesis of the spleen. According to Potter [11], the splenic primordium is solely made up of connective tissue and reticular cells during early fetogenesis, the adult-like spleen attained at the time of birth. In our study, the connective tissue framework started developing from the 13th week of gestation onwards and a network of reticular fibers were visualized in the stained sections.

According to Hamilton et al. [5], splenic condensation starts in the early weeks of gestation and becomes arranged into anastomosing trabeculae as gestation progresses. Later on, trabecular columns produce reticular fibers which lay the foundation of the connective tissue framework of the spleen. During histological changes, the splenic artery opens into spaces called sinusoids, devoid of the endothelial lining. Some of the lining cells become specialized to form a part of the reticuloendothelial system.

In the present study, the formation of splenic lobules was seen in the early developmental stage. A lightly stained "island" with a central artery was seen in the mesenchymal reticulum. Large cells with lightly stained nuclei were arranged around the blood vessel. Frequently, veins were associated with trabeculae within the forming red pulp. The lightly stained "islands" around the central artery became more prominent and surrounded by the forming red pulp with numerous venous sinuses at a later stage. Mesenchymal condensation forming the connective tissue and the developing blood vessels were noted in the 13th and 14th weeks of gestation, which was consistent with Vellguth et al. [12] and Holkunde and Sakahare [13]. According to Copenhaver et al. [9], white pulp showed enlargement in the second trimester, however, obvious splenic nodules with germinal center appeared in the late fetal stage or after birth.

Satoh et al. [14] described the antigenic heterogeneity of the reticular framework, PALS, and marginal zone (MZ) of the white pulp of the fetal spleen of various gestational ages and concluded that the development of the heterogeneity is related to the ontogeny of the PALS, lymphoid follicle, and MZ. Namikawa et al. [15] studied the ontogenic development of lymphoid and non-lymphoid cells in a human splenic white pulp with immunoperoxidase technique, at around 14 weeks of gestation. The primitive white pulp appeared as small accumulations of lymphocytes around arterioles, mainly composed of B1 antigen-positive B cells.

In the present study, splenic lobules were seen to form the primary vascular reticulum in the initial stage, followed by the blood vessels developing in the trabeculae without distinct lobules. The emergence of periarteriolar lymphoid colonization indicated the formation of white pulp. Later on, white pulp gradually grew while red pulp seemed to stop growing further. In the 24th week, white pulp occupied about 50% of the organ volume, whereas, in the postnatal spleen proportion changed, the red pulp made up more than 80% of organ volume [16]. These findings indicate alternative proliferation phases of red and white pulp [3,12] in the splenic primordium of the developing spleen.

The neovascularization of the human fetal spleen which can be grafted in splenic transplantation is crucial, as angiogenesis is mediated by fibroblast growth factors (FGF) mediated signaling pathway by stimulation of hedgehog proteins like angiotensin II. The vasculogenesis will promote the acceptance of the transplanted spleen, in patients with splenectomy. [17]

Our study also showed lymphoid colonization around the arterioles thus forming white pulp which appeared more in the first week of gestation, while in the post-natal spleen, the red pulp is made up of more than 80% volume. This lymphoid tissue found more in human fetuses will decrease the chances of graft versus host disease as the VEGF-B proteins (vascular endothelial growth factor) is responsible for the lymphocyte infiltration in splenic primordium [18].

To date, the only in clinical trials approach of transplantation available is the transplantation of the mesenchymal stem cells but such allograft causes more graft rejection as they are cultured in vivo medium in exogenous models, so the findings of the current study will enhance the literature finding of earlier researchers regarding the future implications of human fetal splenic transplantation, in view of the appearance of angiogenesis and lymphoid aggregation in splenic primordium [19].

Limitations

Further studies on a large sample are required to give a significant reference dataset about the cytoarchitecture of the spleen during development. The mass spectrophotometry to compare the cytoarchitecture of the normal fetal spleen with pathological one and micrometry by image processing program such as Image J Software has to be done to show the number of immunopositivity cells in the field. Also, the grading of the immunostaining has to be performed on a sliding scale of 1+ to 4+ according to the percentage of reactive cells.

## Conclusions

The organizational changes in the splenic primordium, and microstructure of the spleen during fetogenesis are very crucial to achieve the adult morphology in feature. Histogenesis of the fetal spleen follows a multistep process depending upon the gestational age. Any deviation from normalcy may lead to structural and functional abnormality later in life. The findings on the ultrastructural organization of the splenic primordium of the fetal spleen of various weeks of gestation will be beneficial in segmental resection of the spleen as function-preserving therapy in pediatric rupture of the spleen and transplantation surgery among the pediatric age group.
